# Lumbar Ureteral Stenosis due to Endometriosis: Our Experience and Review of the Literature

**DOI:** 10.1155/2013/812475

**Published:** 2013-05-02

**Authors:** Salvatore Butticè, Antonio Simone Laganà, Valeria Barresi, Antonino Inferrera, Giuseppe Mucciardi, Antonina Di Benedetto, Carmela Emanuela D'Amico, Carlo Magno

**Affiliations:** ^1^Unit of Urology, Department of Human Pathology, University of Messina, Via C. Valeria 1, 98125 Messina, Italy; ^2^Unit of Gynecology and Obstetrics, Department of Pediatric, Gynecological, Microbiological and Biomedical Sciences, University of Messina, Via C. Valeria 1, 98125 Messina, Italy; ^3^Unit of Pathological Anatomy, Department of Human Pathology, University of Messina, Via C. Valeria 1, 98125 Messina, Italy

## Abstract

Endometriosis is a chronic gynaecological disorder characterized by the presence of endometrial tissue outside the uterus. The disease most often affects the ovaries, uterine ligaments, fallopian tubes, and cervical-vaginal region. Urinary tract involvement is rare, accounting for around 1%-2% of all cases, of which 84% are in the bladder. We report a case of isolated lumbar ureteral stenosis due to endometriosis in a 37-year-old patient. The patient came to our observation complaining from lumbar back pain and presented with severe fever. The urological examination found monolateral left positive sign of Giordano. Blood tests evidenced marked lymphocytosis and increased valued of C-reactive protein. Urologic ultrasound showed hydronephrosis of first degree in the left kidney and absence of images related to stones bilaterally. Uro-CT scan evidenced ureteral stenosis at the transition between the iliac and pelvic tracts. We addressed the patient to surgery, and performed laparoscopic excision of the paraureteral bulk, endoscopic mechanical ureteral dilation, and stenting. The histological examination evidenced glandular structures lined by simple epithelium and surrounded by stroma. Immunohistochemical test of the glandular epithelium showed positivity for estrogen and progesterone receptors and moreover stromal cells were positive for CD10. The finding suggested a very rare diagnosis of isolated lumbar ureteral endometriosis.

## 1. Introduction

Endometriosis is a disorder characterised by the ectopic presence and growth of functional endometrial tissue, glands, and stroma, outside the uterus [1, 2].

It is classified depending on the number, size, and superficial and/or deep location of endometrial implants, plaques, endometriomas and/or adhesions, as follows: stage I (minimal, 1–5 points), stage II (mild, 6–15 points), stage III (moderate, 16–40 points), and stage IV (severe >40 points), following the revised American Society for Reproductive Medicine classification for Endometriosis (American Society for Reproductive Medicine, 1996) [3].

Approximately 10% of women in reproductive age are estimated to be affected by this disease [4, 5]. Common symptoms are acute or chronic pelvic pain and abnormal bleeding [6].

Pelvic pain could be expressed in a wide range combination of type, such as dysmenorrhea, dyspareunia, dysuria, dyschezia, and nonmenstrual chronic pelvic-abdominal muscle pain [7].

As is suggested by many authors [8, 9], the risk of endometriosis appears to increase for reproductive health factors that may relate to increased exposure to menstruation (i.e., shorter cycle length, longer duration of flow, or reduced parity). The risk appears to decrease for personal habits that may relate to decreased estrogen levels (i.e., smoking and exercise).

The disease most often affects the ovaries (up to 88% of all cases), uterine ligaments, fallopian tubes, rectum, cervical-vaginal region, and urinary tract. Urinary tract involvement is rare accounting 1%-2% of all cases [10], of which 84% are found in the bladder [11].

However, endometriosis can be encountered in other abdominal organs such as the liver, pancreas, intestinal tract, spleen [12], gallbladder [13], the abdominal wall, the navel [14] nasal mucosa [15], or central nervous system [16].

We report a case of isolated lumbar ureteral stenosis due to endometriosis in a 37-year-old female patient.

## 2. Case Presentation

A 37-year-old female patient presented with lumbar pack pain, and severe fever. She had a past medical history of recurrent renal-ureteric colics accompanied by fever, pelvic pain and meteoric bowels and multiple drugs allergy (ciprofloxacin, third-generation cephalosporin, and nonsteroid anti-inflammatory drugs). She reported the first menstruation when she was 10 years old, and following regular menses. Moreover she underwent cesarean section for fetal indication (fetal distress) during her only pregnancy. The urological examination found monolateral left positive sign of Giordano, left kidney area, and costovertebral angle tender to palpation. The ureteral trigger points on the left side were positive to deep palpation and the abdomen was painful but tractable.

Blood test showed neutrophilia and urine analysis showed >1.000.000 of colony forming units of *E. coli*. 

Antibiotic therapy with amoxicillin + clavulanic acid 1 gr × 2/die e.v. and corticosteroid 4 mg/die i.m. was administrated.

**Figure 1:**
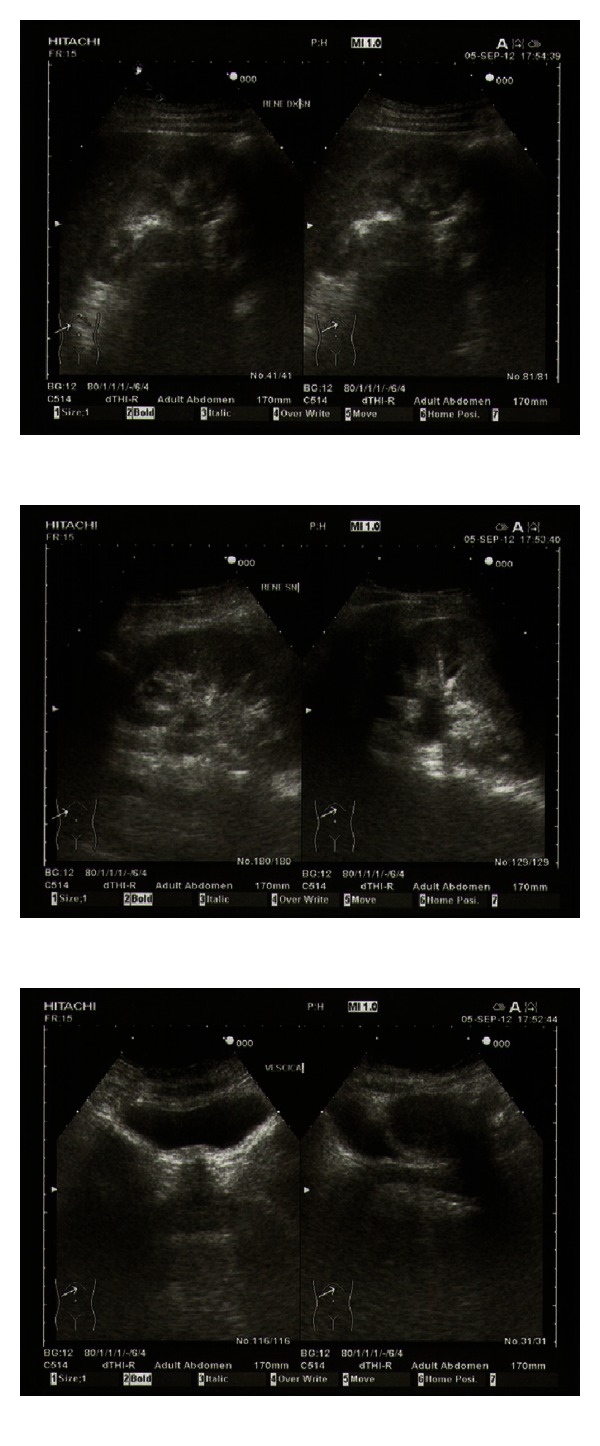
Left kidney of normal size, with increased thickness and parenchymal echogenicity due likely to an inflammatory process, with hydronephrosis of first degree.

**Figure 2:**
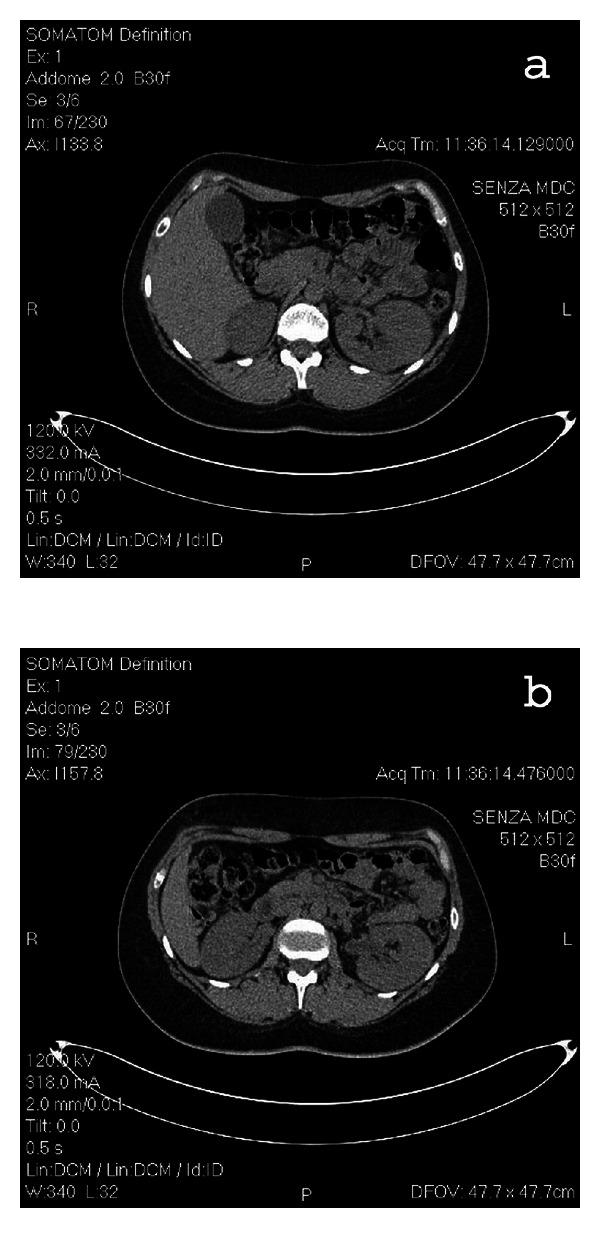
Uro-CT: (a) and (b) confirmation of the first degree hydronephrosis of the left kidney.

Urologic ultrasound (Figure 1) showed left first degree hydronephrosis and the Uro-CT (Figure 2) scan confirmed the first degree hydronephrosis of the left kidney and showed a 15 mm long ureteral stenosis at the transition between the iliac and pelvic tracts.

**Figure 3:**
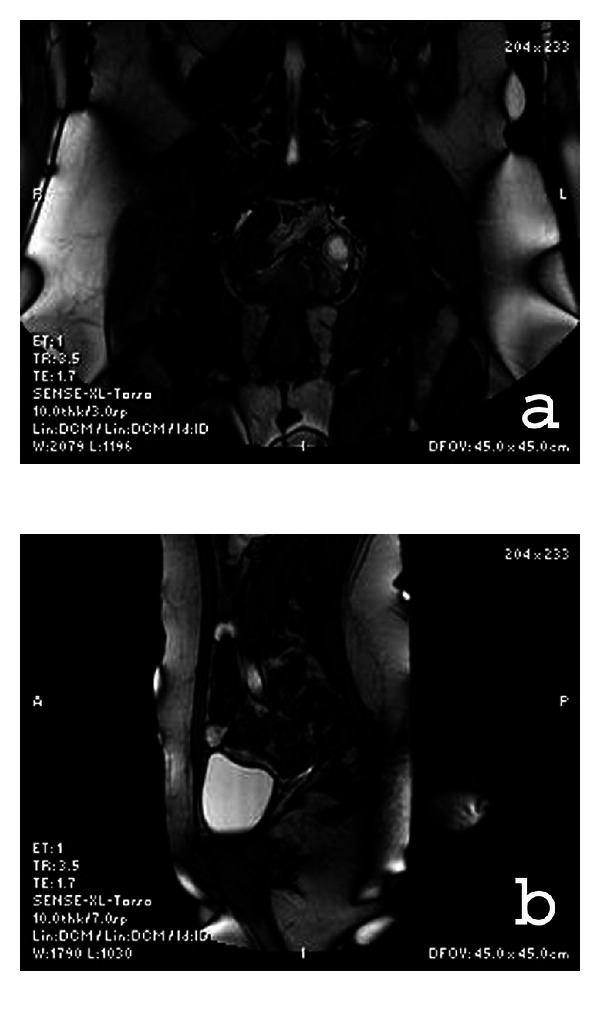
Uro-MRI: (a) and (b) ureteral hyperintense solid bulk below the bifurcation of the left common iliac artery.

Further investigation was done by Uro-MRI (Figure 3) which showed a ureteral hyperintense solid bulk of 12 mm below the bifurcation of the left common iliac artery. 

Suspecting a rare form of endometriosis and according to gynaecology consultant, we performed dosage of tumour markers and hormonal levels, which showed the values reported in Table 1, with a detected increase only in 17 *β* estradiol value.

We addressed the patient to laparoscopic surgery, debulking the endometriotic-like tissue. A contemporary ureteroscopy and ureteral stenting was performed.

Histopathological findings suggested a diagnosis of endometriosis.

**Figure 4:**
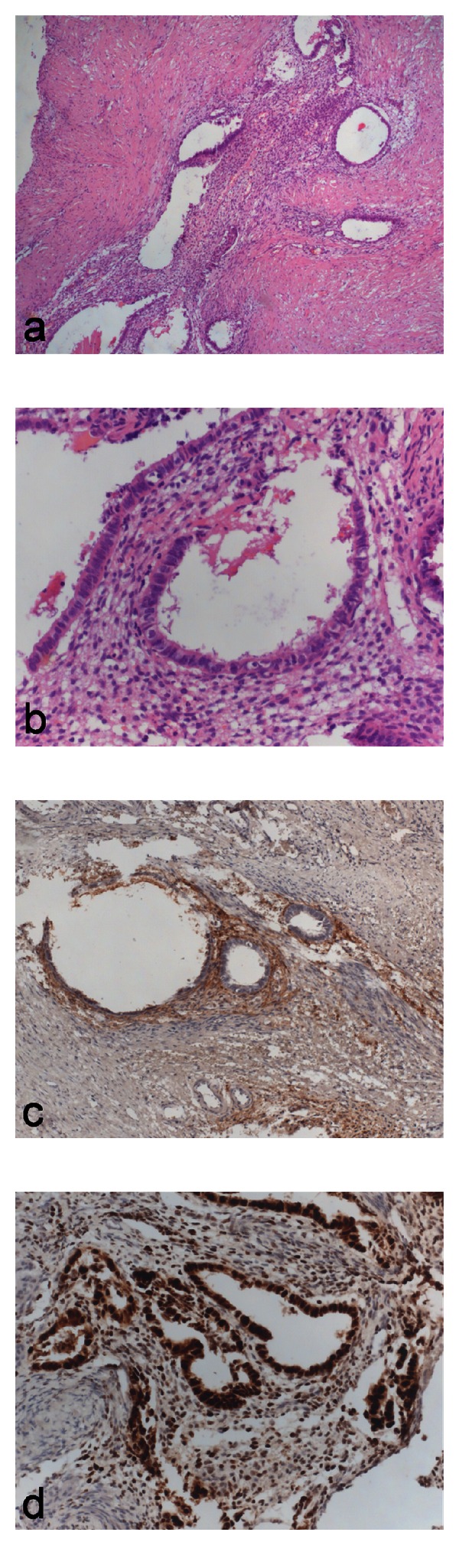
(a) Endometrial-type glands embedded in an endometrial-like stroma were evident within muscular tissue (haematoxylin and eosin stain; original magnification, ×100). (b) Higher magnification of the glands, showing cylindric epithelium lining the glands (haematoxylin and eosin stain; original magnification, ×200). (c) CD10 stain in the endometrial stroma (CD10 stain; original magnification, ×100). (d) Nuclear staining for estrogen receptor in the epithelial and stromal cells of the endometriotic focus (estrogen receptor stain; original magnification, ×200).

Macroscopically, the resected specimen was 1,5 cm in size. It was formalin fixed, paraffin embedded, and cut into 4 *μ*m sections for the histological examination with haematoxylin and eosin stain. Microscopically, variably sized endometrial-type glands lined by a columnar epithelium embedded in an endometrial-like stroma were evident within muscular tissue (Figures 4(a) and 4(b)). Immunohistochemistry demonstrated nuclear staining for estrogen and progesterone receptors (ER and PR) in the glands as well as in the endometrial stroma (Figure 4(d)). Also, CD10 stain was diffusely found in the endometrial-like stroma (Figure 4(c)).

The patient was discharged from hospital in 4 days postoperatively. Ultrasonography and blood examinations 15 days postoperatively were all within normal range. Stent removal was performed 3 months after surgery. At ultrasound control hydronephrosis had regressed completely.

## 3. Discussion

Aetiopathogenesis of endometriosis still remains controversial; immune, hormonal, genetics, and environmental factors seem to be involved. Among the several theories that have been proposed to explain the pathogenesis of the disease, the most popular is that proposed by Sampson in 1927 [17].

According to this theory, during retrograde menstruation, eutopic endometrial cells reflux throughout the tubes to the peritoneal cavity, adhere to the peritoneal wall, proliferate, and form endometriotic lesions. Although so far it was not disproved, this theory seems to be not definitive, because retrograde menstruation could be observed in 90% of endometriosis-free women in reproductive age with pervious fallopian tubes without causing the disease. Another theory postulates that endometriotic foci could arise from endometrial cells that enter in the uterine venous or lymphatic circulation; other Authors [18–20], on the contrary, suggest that endometriosis may derive from a displacement of the primitive tissue that gives rise to endometrial cells, caused by incorrect reproductive tract organogenesis (embryonic derivation theory).

There is also the possibility that the disease originates from a process of metaplasia of cells of the visceral and abdominal peritoneums (coelomic origin), as a result of continuous pacing by yet unknown stimuli [21].

In the case that we have previously described, we hypothesize that endometriotic focus on left lumbar ureteral may be derived from endometrial debris refluxed by retrograde menstruation, or via uterine vessel circulation. According to this way of developing, it is quite uncommon that we have not found any other endometriotic implants in the peritoneum or in other pelvic sites, nor fibrosis and adhesions between pelvic organs. For this reason, another possible hypothesis to explain the isolated left lumbar ureteral endometriosis (that we observed) is that it could be due to Müllerian-derived progenitor cells that, after certain stimuli, evolved to form the typical implant.

Depending on location and extension of endometriotic implant, we could summarily divide among superficial peritoneal, ovarian, and deep infiltrating endometriosis (DIE); this last form, characterized by infiltration for more than 5 mm beyond the wall of the pelvic peritoneum, usually involving uterosacral ligaments, rectovaginal spaces, the upper third of the posterior wall of the vagina, the bowel, and urinary tract [22] is reported by Nezhat et al. [23]

Our case is according to that described by Traşcă et al. [24], because we observed nonspecific symptoms, pseudotumoral development, and impossibility to establish a preoperative aetiological diagnosis. The peculiarity of our case is that the endometriotic implants involve chiefly the lumbar ureter, without any other location; this is very rare considering that ureteral endometriosis usually involves the pelvic tract of ureter. Finally, endometriosis should be considered as a cause of monolateral ureterohydronephrosis without evidence of stones in a female patient in reproductive age, even if it will be a remote and rare occurrence.

## Figures and Tables

**Table 1:** Tumour markers and hormonal levels.

Analyte	Value	Normal range
Tumour markers values		

CA 125 (cancer antigen 125)	21,84 UI/mL	0–33 UI/mL
CA 19.9 (cancer antigen 19.9)	8,25 UI/mL	0–40 UI/mL
CEA (carcinoembryonic antigen)	0,46 ug/mL	Nonsmoker (as was the patient): 0–3 ug/mL.
AFP (alpha-fetoprotein)	0,97 ng/mL	0–7,5 ng/mL

Hormonal levels		

FSH (follicle stimulating hormone)	4.46 mIU/mL	Follicular phase (as was the patient): 3,5–12,5 mIU/mL
LH (luteinizing hormone)	3.04 mIU/mL	Follicular phase (as was the patient): 2,4–12,6 mIU/mL
E2 (17 *β* estradiol)	227 pg/mL	Follicular phase (as was the patient): 12,5–166 pg/mL
PG (progesterone)	0,70 ng/mL	Follicular phase (as was the patient): 0,2–1,5 ng/mL
*β*-HCG (*β*-human chorionic gonadotropin)	Negative	

## References

[1] Baldi A, Campioni M, Signorile PG (2008). Endometriosis: pathogenesis, diagnosis, therapy and association with cancer. *Oncology Reports*.

[2] Bulun SE (2009). Endometriosis. *The New England Journal of Medicine*.

[3] American Society for Reproductive Medicine (1997). Revised American Society for Reproductive Medicine classification of endometriosis: 1996. *Fertility and Sterility*.

[4] Huhtinen K, Perheentupa A, Poutanen M, Heikinheimo O (2011). Pathogenesis of endometriosis. *Duodecim*.

[5] Marana R, Lecca A, Biscione A, Muzii EL (2012). Endometriosis: the gynecologist's opinion. *Urologia*.

[6] Viganò P, Parazzini F, Somigliana E, Vercellini P (2004). Endometriosis: epidemiology and aetiological factors. *Best Practice and Research: Clinical Obstetrics and Gynaecology*.

[7] Stratton P, Berkley KJ (2011). Chronic pelvic pain and endometriosis: translational evidence of the relationship and implications. *Human Reproduction Update*.

[8] Eskenazi B, Warner ML (1997). Epidemiology of endometriosis. *Obstetrics and Gynecology Clinics of North America*.

[9] Viganò P, Parazzini F, Somigliana E, Vercellini P (2004). Endometriosis: epidemiology and aetiological factors. *Best Practice and Research: Clinical Obstetrics and Gynaecology*.

[10] Westney OL, Amundsen CL, McGuire EJ (2000). Bladder endometriosis: conservative management. *Journal of Urology*.

[11] Shook TE, Nyberg LM (1988). Endometriosis of the urinary tract. *Urology*.

[12] Sinder C, Dochat GR, Wentsler NE (1965). Splenoendometriosis. *American Journal of Obstetrics and Gynecology*.

[13] Saadat-Gilani K, Bechmann L, Frilling A, Gerken G, Canbay A (2007). Gallbladder endometriosis as a cause of occult bleeding. *World Journal of Gastroenterology*.

[14] Kyamidis K, Lora V, Kanitakis J (2011). Spontaneous cutaneous umbilical endometriosis: report of a new case with immunohistochemical study and literature review. *Dermatology Online Journal*.

[15] Laghzaoui O, Laghzaoui M (2001). Nasal endometriosis: apropos of 1 case. *Journal de Gynécologie, Obstétrique et Biologie de la Reproduction*.

[16] Ichida M, Gomi A, Hiranouchi N (1993). A case of cerebral endometriosis causing catamenial epilepsy. *Neurology*.

[17] Sampson JA (1927). Peritoneal endometriosis due to menstrual dissemination of endometrial tissue into the peritoneal cavity. *American Journal of Obstetrics & Gynecology*.

[18] Signorile PG, Baldi F, Bussani R, D’Armiento M, de Falco M, Baldi A (2009). Ectopic endometrium in human foetuses is a common event and sustains the theory of müllerianosis in the pathogenesis of endometriosis, a disease that predisposes to cancer. *Journal of Experimental and Clinical Cancer Research*.

[19] Signorile PG, Baldi F, Bussani R (2010). New evidence of the presence of endometriosis in the human fetus. *Reproductive BioMedicine Online*.

[20] Signorile PG, Baldi F, Bussani R (2012). Embryologic origin of endometriosis: analysis of 101 human female fetuses. *Journal of Cellular Physiology*.

[21] Gruenwald P (1942). Origin of endometriosis from the mesenchyme of the celomic walls. *American Journal of Obstetrics and Gynecology*.

[22] Chapron C, Chopin N, Borghese B (2006). Deeply infiltrating endometriosis: pathogenetic implications of the anatomical distribution. *Human Reproduction*.

[23] Nezhat CH, Malik S, Osias J, Nezhat F, Nezhat C (2002). Laparoscopic management of 15 patients with infiltrating endometriosis of the bladder and a case of primary intravesical endometrioid adenosarcoma. *Fertility and Sterility*.

[24] Traşcă ET, Traşcă E, Tiţu A, Riza ML, Busuioc I (2012). Ureteral stenosis due to endometriosis. *Romanian Journal of Morphology and Embryology*.

